# Supplementary material for: Exploring the Perspectives of Canadian Clinicians Regarding Digitally Delivered Psychotherapies Utilized for Trauma-Affected Populations
Source: Int J Environ Res Public Health. 2025 Jan 9;22(1):81. doi: 10.3390/ijerph22010081 (PMC11765287; doi:10.3390/ijerph22010081)
Supplement: Supplementary file 1 [file ijerph-22-00081-s001.zip › ijerph-3313437-supplementary.pdf]

File S1. Copies of Alberta Quality Matrix for Health, Unified Theory of Acceptance and Use of Technology, and Surveys

Figure 1. Copy of the Alberta Quality Matrix for Health. (Health Quality Council of Alberta, 2017)

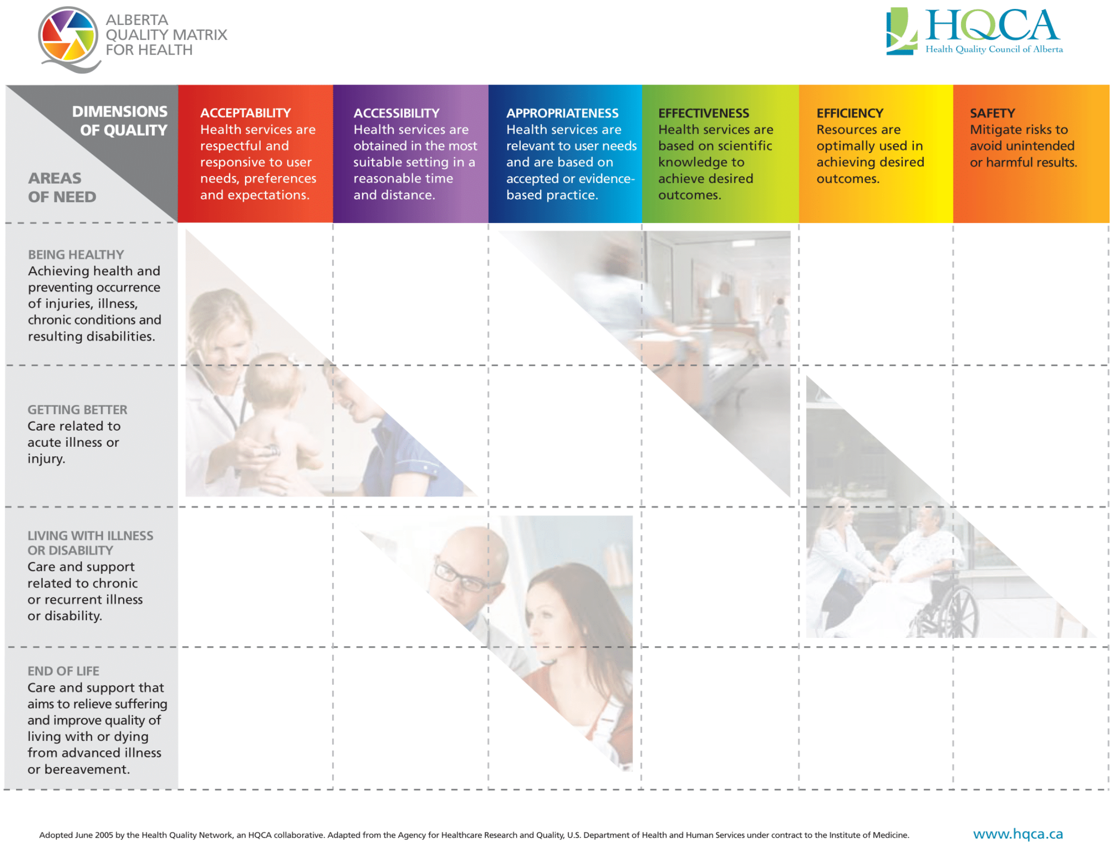

Quality Matrix for Health Questionnaire - Digitally Delivered Trauma Therapy Clinician Version

Instructions

The Quality Matrix for Health Questionnaire Tool is used to evaluate the quality-of-service delivery based on 10 criteria, which appear below. For each criterion, please provide a rating for in person and digital delivery of trauma therapy using a scale of 1 (low) to 7 (high) and <u>offer comments/an explanation ONLY if you wish.</u>

1. Ease of use refers to the degree to which it can be used without much effort.

In person: : 1---2---3---4---5---6---7
Digital: 1---2---3---4---5---6---7
Comments/Explanation:

2. Convenience refers to the degree to which it saves or simplifies work and adds to one's ease or comfort.

In person: : 1---2---3---4---5---6---7
Digital: 1---2---3---4---5---6---7
Comments/Explanation:

3. Acceptability refers to whether it was respectful and responsive to user needs, preferences and expectations.

In person: : 1---2---3---4---5---6---7
Digital: 1---2---3---4---5---6---7
Comments/Explanation:

4. Practicality refers to how feasible it is.

In person: : 1---2---3---4---5---6---7
Digital: 1---2---3---4---5---6---7
Comments/Explanation:

5. Accessibility refers to whether it is delivered in a suitable setting in a reasonable time and distance

In person: : 1---2---3---4---5---6---7
Digital: 1---2---3---4---5---6---7
Comments/Explanation:

6. Appropriateness refers to whether it is relevant to patient needs.

In person: : 1---2---3---4---5---6---7
Digital: 1---2---3---4---5---6---7
Comments/Explanation:

7. Effectiveness refers to whether it helps to achieve desired outcomes.

In person: : 1---2---3---4---5---6---7
Digital: 1---2---3---4---5---6---7
Comments/Explanation:

8. Efficiency refers to whether it uses resources optimally to achieve desired outcomes.

In person: : 1---2---3---4---5---6---7
Digital: 1---2---3---4---5---6---7
Comments/Explanation:

9. Safety refers to whether it reduces risks and avoids unintended or harmful results.

In person: : 1---2---3---4---5---6---7
Digital: 1---2---3---4---5---6---7
Comments/Explanation:

10. Fit refers to how well it aligns with initiatives, structures, and supports within clinics/Alberta Health Services, as well as the priorities of patients and clinicians.

In person: : 1---2---3---4---5---6---7
Digital: 1---2---3---4---5---6---7
Comments/Explanation:

General Paragraph Text Boxes:

In light of the above factors 1-10 regarding the digital delivery of trauma therapy for you, your organization and patients:

What works well and for whom? Please explain.

What doesn't work well and for whom? Please explain.

What lessons were learned from the rapid transition to digital delivery?

What would you recommend? Please explain.

Figure 2. Copy of the Unified Theory of Acceptance and Use of Technology (UTAUT) Model. (Venkatesh et al., 2003)

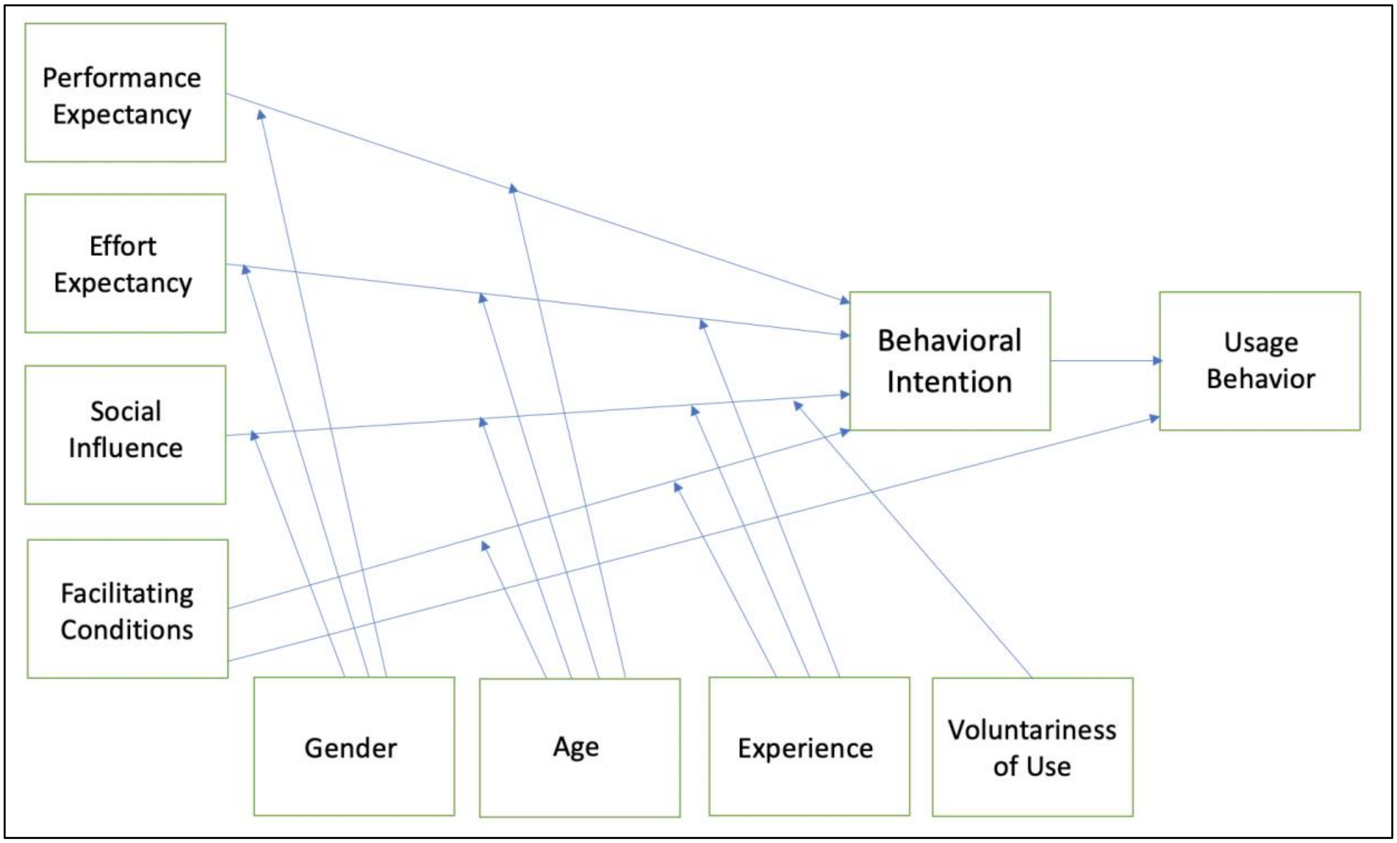

Digital Delivery of Mental Health Therapy – For Clinicians WITH Experience with Digital Therapy							
The purpose of this survey is to gather data on what factors affect client use of a digital system to deliver mental health therapy during the therapy sessions. Please indicate your level of agreement with each item. Mark only one X in a box per item. In the questions below, "digital delivery of therapy" means doing mental health therapy sessions remotely, with the therapist and patient at different locations but talking with each other over the internet or a telephone.							
**Item**	**Strongly disagree (1)**	**Disagree (2)**	**Slightly disagree (3)**	**Neither agree nor disagree (4)**	**Slightly agree (5)**	**Agree (6)**	**Strongly agree (7)**
1. Using digital delivery of therapy improved the mental health of my patients (PE-PU1)							
2. Using digital delivery of therapy had a positive effect on the mental health of my patients (PE-JF2)							
3. Using digital delivery of therapy has improved the quality of life of my patients (PE-JF3)							

4. Interacting with the system used for digital delivery of therapy was easy for me (EE-EU1)							
5. Interacting with a digital system to deliver therapy was clear and understandable (EE-EU2)							
6. The system used for digital delivery of therapy was easy to use (EE-PEU3)							
7. People who are important to me think that I should be involved in using digital delivery of therapy (SI-SN1)							
8. I used digital delivery of therapy because my colleagues used it too (SI-SF2)							
9. In general, my organization supported my use of digital delivery of therapy (SI-SF3).							
10. Guidance was available to me during my interaction with the system used for digital delivery of therapy (FC-FC1)							
11. Specialized instruction on how to use the system used for digital delivery of therapy was available to me (FC-FC2)							
12. A specific person (or group of people) was available to assist with any difficulties with digital delivery of therapy (FC-FC2)							

13. I am willing to continue using digital delivery of therapy in the future (BI1)							
14. I plan to continue using digital delivery of therapy in the future (BI2)							
15. I predict I will use a digital delivery of therapy in the future (BI3)							
16. I used digital delivery of therapy to explore an alternative way to improve the quality of life of my patients (U1)							
17. I used digital delivery of therapy to explore an alternative way improve the mental health of my patients (U2)							
18. I used digital delivery of therapy to explore an alternative way of having positive effect on the mental health of my patients (U3)							

References

Health Quality Council of Alberta. Healthcare Quality and Safety Management: A Framework for Alberta. Report. Health Quality Council of Alberta, Calgary, AB, Canada, 2017; 52p.

Venkatesh, V.; Morris, M.G.; Davis, G.B.; Davis, F.D. User acceptance of information technology: toward a unified view. *MIS Quarterly*. **2003**, *27*(3), 425-478. doi: 10.2307/30036540.

File S2. Semi-Structured Interview and Focus Group Script

Semi-Structured Interview and Focus Group Questions – **Mental Health Clinicians**

COVID-19 Physical Distancing, Virtual Delivery of Trauma Therapies to Trauma-Affected Populations

This interview/focus group follows an iterative process (additional questions may be asked based on the answers to the questions within this guide).

1. **Introduction:** Can you tell us about your organization and your role within the organization?

2. What do you know about online digital mental health interventions?
    a. What do you know about DH for TAPs?

3. **Current State and Needs Analysis**:
    a. What has the experience been like regarding the rapid transition to remote digital health (DH) mental health care?
    b. Broadly speaking and in a perfect situation, what would the use of DH look like and why?
    c. Were there any unexpected outcomes? Positive or Negative?
    d. What information/training would you find helpful to increase and better engage your use of online DMH interventions with TAPs in your clinical practice?
    e. Are there individuals who have been disproportionately affected by COVID-19 and accessing services? Are there opportunities to address these gaps with D-MH?

4. **Barriers / Supports / Facilitators**
    a. What parts of the transition to DH was most challenging and why?
    b. What parts of the transition to DH were the easiest and why?

5. **Technological Acceptance**
    a. Have there been any logistic or security issues (i.e., firewalls, compatibility, or policies) that have challenged the ability to use DH?
    b. What has the feasibility been like?
    c. What has the technological acceptance been like?
    d. What has the feedback been like from clinicians? Frontline personnel? Care recipients? Persons with lived experience? Care givers? IT specialists?

6. **Resourcing**
    a. What resources did your organization/clinic need to bring in to support DH?
    b. Have those resources' needs changed over time?
    c. Was there a financial cost associated with requiring those resources? If yes, what has been the impact of that financial burden?
    d. Did your clinicians require additional administrative or technical support to implement DH?

7. **Methods of Delivery** – What has been the best way to deliver DH trauma therapy for TAPs? Telehealth? Videoconferencing? Apps?
    a. Do you have some thoughts about how the D-MH platform might be used in both intake assessments and ongoing treatment planning in your daily practice?

8. **Clinical Effectiveness:**
    a. Has use of DH for trauma therapy been effective from the perspective of persons with lived experience?
    b. Were you satisfied with the care? Explain.
    c. Are there certain trauma modalities which are better or worse suited for DH?
    d. Have you had to adapt any practices associated with trauma modalities?
    e. Are there certain patients which you have found are better or worse suited for DH?
    f. Any thoughts about privacy, confidentiality, and patient safety when using D-MH? Any other professional or ethical issues?
    g. Do you perceive any professional/ethical issues related to D-MH?
    h. Do you currently have a way of measuring progress or change for TAPs in your services? What are the key metrics? How could D-MH help to track and demonstrate progress?

9. **Implementation Needs** - What would need to change to better integrate DH in practice?
    a. **What** will/should stay the same/evolve?

b. **What** would successful implementation look like and why?
c. **Who** - should help mobilize use of DH?
d. **Scaling and sustainability** – is the ongoing use of DH sustainable? Ought the use of DH continue after COVID-19? Describe and explain

File S3. Statistical Test Summaries

Table 1. Clinician Alberta Quality Matrix for Health median score paired sample Wilcoxon signed-rank test summary comparing median scores for digital delivery vs in-person therapy.

	Null Hypothesis	Test	Sig.[a,b]	Decision
1	The median of EoU_C equals .00.	One-Sample Wilcoxon Signed Rank Test	.943	Retain the null hypothesis.
2	The median of Conv_C equals .00.	One-Sample Wilcoxon Signed Rank Test	.681	Retain the null hypothesis.
3	The median of Acc_C equals .00.	One-Sample Wilcoxon Signed Rank Test	.952	Retain the null hypothesis.
4	The median of Prac_C equals .00.	One-Sample Wilcoxon Signed Rank Test	.114	Retain the null hypothesis.
5	The median of Asb_C equals .00.	One-Sample Wilcoxon Signed Rank Test	.020	Reject the null hypothesis.
6	The median of App_C equals .00.	One-Sample Wilcoxon Signed Rank Test	.046	Reject the null hypothesis.
7	The median of Eff_C equals .00.	One-Sample Wilcoxon Signed Rank Test	.194	Retain the null hypothesis.
8	The median of Efc_C equals .00.	One-Sample Wilcoxon Signed Rank Test	.785	Retain the null hypothesis.
9	The median of Saf_C equals .00.	One-Sample Wilcoxon Signed Rank Test	.492	Retain the null hypothesis.
10	The median of Fit_C equals .00.	One-Sample Wilcoxon Signed Rank Test	.395	Retain the null hypothesis.

a. The significance level is .050.

b. Asymptotic significance is displayed.

Table 2. Clinician Unified Theory of Acceptance and Use of Technology one-sample Wilcoxon signed-rank test summary comparing median score and a reference score of 12 (total score of three questions asked based on Likert scale 1-7).

	Null Hypothesis	Test	Sig.[a,b]	Decision
1	The median of utautPE_C equals 12.00.	One-Sample Wilcoxon Signed Rank Test	.003	Reject the null hypothesis.
2	The median of utautEE_C equals 12.00.	One-Sample Wilcoxon Signed Rank Test	.012	Reject the null hypothesis.
3	The median of utautSI_C equals 12.00.	One-Sample Wilcoxon Signed Rank Test	.017	Reject the null hypothesis.
4	The median of utautFC_C equals 12.00.	One-Sample Wilcoxon Signed Rank Test	.593	Retain the null hypothesis.
5	The median of utautBI_C equals 12.00.	One-Sample Wilcoxon Signed Rank Test	.002	Reject the null hypothesis.
6	The median of utautUB_C equals 12.00.	One-Sample Wilcoxon Signed Rank Test	.004	Reject the null hypothesis.

a. The significance level is .050.

b. Asymptotic significance is displayed.

Table 3. Clinician outcome measure score significance following Benjamini-Hochberg procedure to control False Discovery Rate and correct for multiple comparisons.

**Variable**	**K**	**P-value (*significant after correction)**	**Benjamini-Hochberg p-value Correction**
Clinician UTAUT Behavioral Intention	1	0.0020*	0.0031
Clinician UTAUT Performance Expectancy	2	0.0030*	0.0063
Clinician UTAUT Use Behaviour	3	0.0040*	0.0094
Clinician UTAUT Effort Expectancy	4	0.0120*	0.0125
Clinician UTAUT Social Influence	5	0.0170	0.0156
Clinician AQMH Accessibility	6	0.0200	0.0188
Clinician AQMH Appropriateness	7	0.0460	0.0219
Clinician AQMH Practicality	8	0.1140	0.0250
Clinician AQMH Efficiency	9	0.1940	0.0281
Clinician AQMH Fit	10	0.3950	0.0313
Clinician AQMH Safety	11	0.4920	0.0344
Clinician UTAUT Facilitating Conditions	12	0.5930	0.0375
Clinician AQMH Convenience	13	0.6810	0.0406
Clinician AQMH Efficiency	14	0.7850	0.0438
Clinician AQMH Ease of Use	15	0.9430	0.0469
Clinician AQMH Acceptability	16	0.9520	0.0500
